# Contemporary benefit‐harm profile over two decades in primary prophylactic ICD‐therapy

**DOI:** 10.1002/clc.23234

**Published:** 2019-07-17

**Authors:** Thomas Kleemann, Margit Strauss, Kleopatra Kouraki, Eleni Lampropoulou, Andràs Fendt, Nicolas Werner, Ralf Zahn

**Affiliations:** ^1^ Klinikum Ludwigshafen Medizinische Klinik B Ludwigshafen Germany

**Keywords:** defibrillation, ICD complications, ICD shock, implantable cardioverter defibrillator (ICD), primary prophylactic ICD

## Abstract

**Background:**

Implantable cardioverter defibrillator (ICD) was implemented into clinical routine more than 20 years ago. Since then, ICD therapy became standard therapy for primary and secondary prevention of sudden cardiac death in clinical practice.

**Objectives:**

Aim of the study was to evaluate the benefit‐harm profile of contemporary primary prophylactic ICD therapy.

**Methods:**

A total of 1222 consecutive patients of a prospective single‐center ICD‐registry were analyzed who underwent primary prophylactic ICD implantation between 2000 and 2017. Patients were divided into two groups according to the implantation year: 2010‐2017 (group 1, n = 579) and 2000‐2009 (group 2, n = 643).

**Results:**

The rate of estimated appropriate ICD therapy after 8 years was 51% in the 2000s and 42% in the 2010s (*P* < .001). The complication rate changed slightly from 53% to 47% (*P* = .005). This decline was mainly driven by the reduction of inappropriate ICD shocks (30% vs 14%, *P* < .001) whereas the rate of ICD shock lead malfunction and device/ lead infection remained unchanged over time. Nonischemic cardiomyopathy was an independent predictor for ICD complications without benefit of ICD therapy (HR 1.37, 95% CI 1.07‐1.77).

**Conclusion:**

The ICD therapy rate for ventricular arrhythmias in patients with primary prophylactic ICD implantation is decreasing over the last two decades. Complication rate remains high due to an unchanged rate of ICD shock malfunctions and device infections. Nonischemic cardiomyopathy is an independent predictor for ICD complications without benefit of ICD therapy in primary prophylactic ICD‐therapy.

## 1 INTRODUCTION

The implantable cardioverter defibrillator (ICD) therapy was implemented into clinical routine more than 20 years ago. Since then it became standard therapy for primary and secondary prevention of sudden cardiac death in clinical practice. Randomized studies like the Multicenter Automatic Defibrillator Implantation Trial (MADIT) or Sudden Cardiac Death in Heart Failure Trial (SCD‐HeFT) which introduced the ICD therapy for primary prophylactic indication to standard heart failure treatment were conducted in the 90s or beginning of the 2000s.^1^, ^2^ Treatment of heart failure patients in these trials are not comparable with today's clinical state of optimized heart failure treatment. In MADIT or SCD‐HeFT patient's prescription rate of beta‐blocker was 70% and spironolactone 20%, which is low compared to today's expected optimized heart failure treatment.^1^, ^2^, ^3^ Cardiac resynchronization therapy (CRT) which is indicated for up to 50% of ICD patients in registries were not included into the early primary prophylactic prevention studies.^4^ The DANISH trial, a recently published randomized study dealing with the beneficial effect of ICD therapy in nonischemic cardiomyopathy, had a nearly 50% CRT therapy and could not show a benefit of ICD therapy compared to optimized medical treatment.^5^ With the implementation of optimized heart failure therapy in daily clinical practice patients with heart failure today have a higher life expectancy than in the 2000s.^3^, ^6^, ^7^ ICD shock lead performance becomes more relevant with longer patient's life as the ICD shock lead failure increases up to 20% after 8 years of ICD therapy.^8^ Device recalls or lead performance alerts can force the patient to undergo additional revision surgeries that implicates further risks for the patient. For a potential ICD patient in the late 2010s the benefit and harm of ICD therapy might have changed over time during the last decades. Therefore, the present study evaluates the contemporary benefit‐harm profile in patients undergoing primary prophylactic ICD therapy.

## 2 METHODS

### 2.1 Patient characteristics and follow‐up

A total of 1222 of 2378 (51%) patients of the prospective single‐center ICD‐registry Ludwigshafen who underwent ICD implantation between 2000 and 2017 and had a primary prophylactic ICD indication with an ejection fraction (EF) ≤ 40% were included into the present study. The prospective single center ICD‐registry Ludwigshafen has been previously described in detail.^8^ Written informed consent was obtained from all patients. The study was approved by the local ethics committee of the Landesärztekammer Rheinland Pfalz. The implanted devices were manufactured by Medtronic (n = 176), Abbott, former St. Jude Medical (n = 1040), Biotronik (n = 1), Boston Scientific (n = 4) and Sorin (n = 1). All ICDs were implanted in an operating theater. The majority of the ICDs (> 95%) were implanted by 2 operators with long experience of ICD implantation. Most of the ICD‐leads (> 95%) were implanted via a subclavian puncture. The most frequently implanted ICD leads models were: Durata leads (n = 674), Riata leads (n = 327, both Abbott, former St. Jude Medical) and 6944 respectively 6947 Sprint Quattro ICD leads (n = 145, Medtronic). After ICD implantation, all patients visited the defibrillator outpatient clinic every 3 to 6 months as well as in case of any adverse event. Antitachycardia pacing (ATP) or shocks were considered to be appropriate if the triggering rhythm was determined to be ventricular fibrillation (VF) or VT.

Benefit of ICD therapy was defined as appropriate termination of ventricular tachyarrhythmias by ICD. Harm of ICD therapy included perioperative complications related to ICD‐implantation or revision, ICD lead failure or lead malfunction requiring lead revision or termination of ICD therapy, device recalls, inappropriate ICD shocks, ICD pocket problems and device infection. The time to follow‐up was the period up to the most recent follow‐up visit or the time to death. For the present analysis, the maximum follow‐up duration was 8 years. The follow‐up ended in September 2018. In 49 patients, ICD therapy was stopped due to the clinical status of the patient or heart transplantation.

### 2.2 ICD programming

From 2000 to 2005 all patients received two zones of therapy: (a) a VF zone which was defined as an episode of tachycardia with at least 12 beats at a rate of 200 beats per minute or more; in this zone up to six shocks could be delivered per episode; (b) a VT zone with an episode of tachycardia with at least 12 beats at a rate between 167 and 200 beats per minute. Three ATP bursts were followed by up to five shocks if the tachycardia did not terminate previously. In 2005, clinical routine changed. Patients with newly implanted ICD due to primary prophylactic ICD indication received only a VF therapy zone and a monitor zone was programmed for detection of VTs between 167 and 200 beats per minute. If VT was detected in the monitor zone during follow‐up, a VT therapy zone was activated, too. Since 2012, the device programming was adapted according to the MADIT Rit trial, which consisted of a prolonging of the number of beats for detection of VT and VF.^9^

### 2.3 Statistical analysis

The patient population is described by absolute numbers and percentages. The distribution of continuous variables is characterized by means and SD, or medians with upper/lower quartile. Patients were divided into two groups according to the implantation decade: 2010‐2017 (group 1, n = 579) and 2000‐2009 (group 2, n = 643). Categorical variables were compared by using the Pearson chi‐square or Fisher's exact test, as appropriate.

Kaplan‐Meier curves were calculated to compare the different endpoints. Differences were compared by using the log rank test. Cox regression analysis was performed to find independent predictors for benefit or harm from ICD therapy. The following parameters were included into the multivariate analysis: age > 70 years, female gender, EF < 30%, nonischemic heart disease, diabetes, atrial fibrillation, CRT, Riata ICD lead and implantation decade. All P‐values were two‐tailed. A P‐value <.05 was considered to be statistically significant. The tests were performed using SPSS. The authors had full access to the data and take complete responsibility for the integrity of the data. All authors have read and agreed to the manuscript as written.

## 3 RESULTS

### 3.1 Patient characteristics

Baseline clinical data of the patients stratified according to the implantation decade are summarized in Table 1. Patients with ICD‐implantation between 2010 and 2017 (group 1) were more often female, had less often atrial fibrillation or renal failure (Table 1). They were less often treated with digoxin or amiodarone but more often received spironolactone than patients with ICD implantation between 2000 and 2009 (group 2, Table 1). No differences were observed between both groups with regard to age, EF or the underlying cardiac disease.

**Table 1:** Clinical characteristics of patients at ICD implantation

	Group 1: implant 2010‐2017 (n = 579)	Group 2: implant 2000‐2009 (n = 643)
Clinical characteristics		
Age (years)	64 ± 11^a^	63 ± 10
Female sex	20%	16%^b^
EF < 30%	84%	83%
Coronary artery disease	52%	59%
History of AF	30%	36%^b^
Hypertension	78%	77%
Diabetes	33%	35%
COPD	14%	16%
Prior stroke	13%	11%
Renal impairment	23%	30%^b^
Implanted ICD systems		
Single chamber device	24	28%
Dual chamber device	35%	31%
Biventricular device	41%	41%
Medication at discharge		
ACE/ARB/ARNI	94%	97%
Beta blocker	93%	96%
Spironolactone	76%	60%^c^
Digoxin	18%	44%^c^
Diuretics	81%	85%
Amiodarone	4.1%	6.8%

ACE, angiotensin converting enzyme; AF, atrial fibrillation; ARB, angiotensin receptor antagonist; ARNI, angiotensin receptor‐neprilysin inhibitor; COPD, chronic obstructive pulmonary disease; EF, ejection fraction; ICD, implantable cardioverter defibrillator; n.s., nonsignificant.

a Values are presented as mean ± SD.

b *P* < .05.

c *P* < .001.

### 3.2 Benefit and harm of ICD therapy during follow‐up

**Figure 1:**
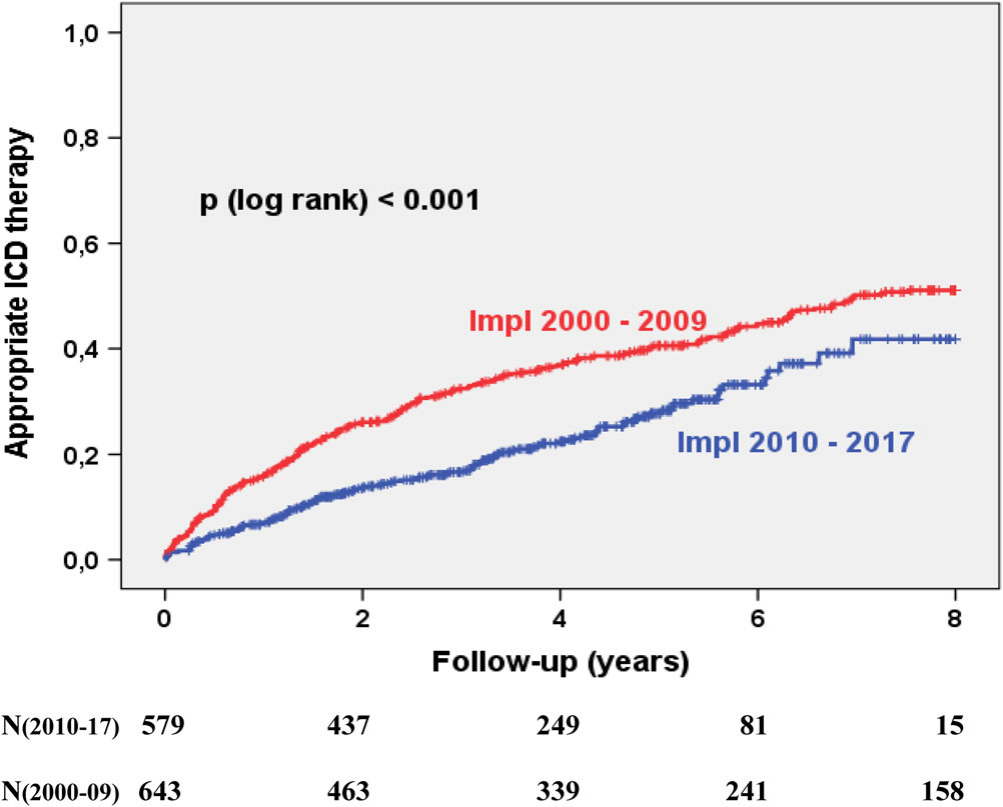
Kaplan‐Meier curves showing the incidence of appropriate ICD therapy. Patients were stratified according to the implantation decade. ICD, implantable cardioverter defibrillator

Patients with implantation between 2010 and 2017 (group 1) had a 12% lower 8 year mortality than patients implanted in the 2000s (27% vs 39%, *P* < .001). The rate of estimated appropriate ICD therapy after 8 years was 51% in the 2000s and 42% in the 2010s (*P* < .001, Figure 1). This decline was observed in all subgroups: VT/VF shocks, VF shocks, VT shocks and ATP for VT.

**Figure 2:**
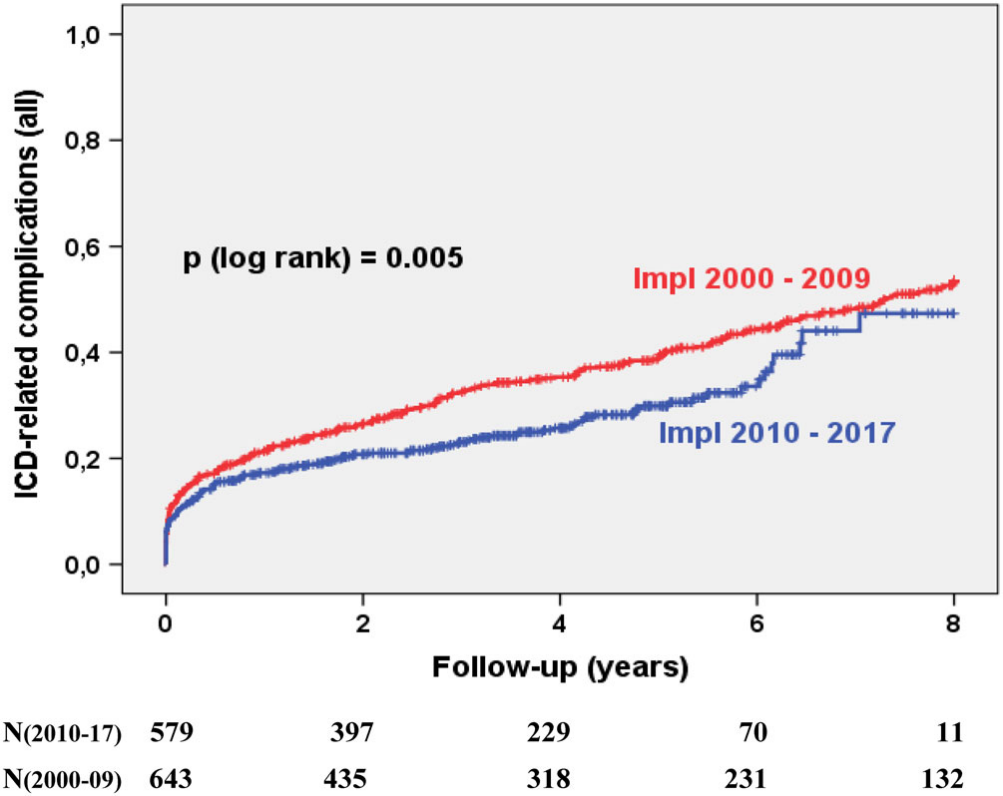
Kaplan‐Meier curves showing the incidence of ICD‐related complications. Patients were stratified according to the implantation decade. ICD, implantable cardioverter defibrillator

**Figure 3:**
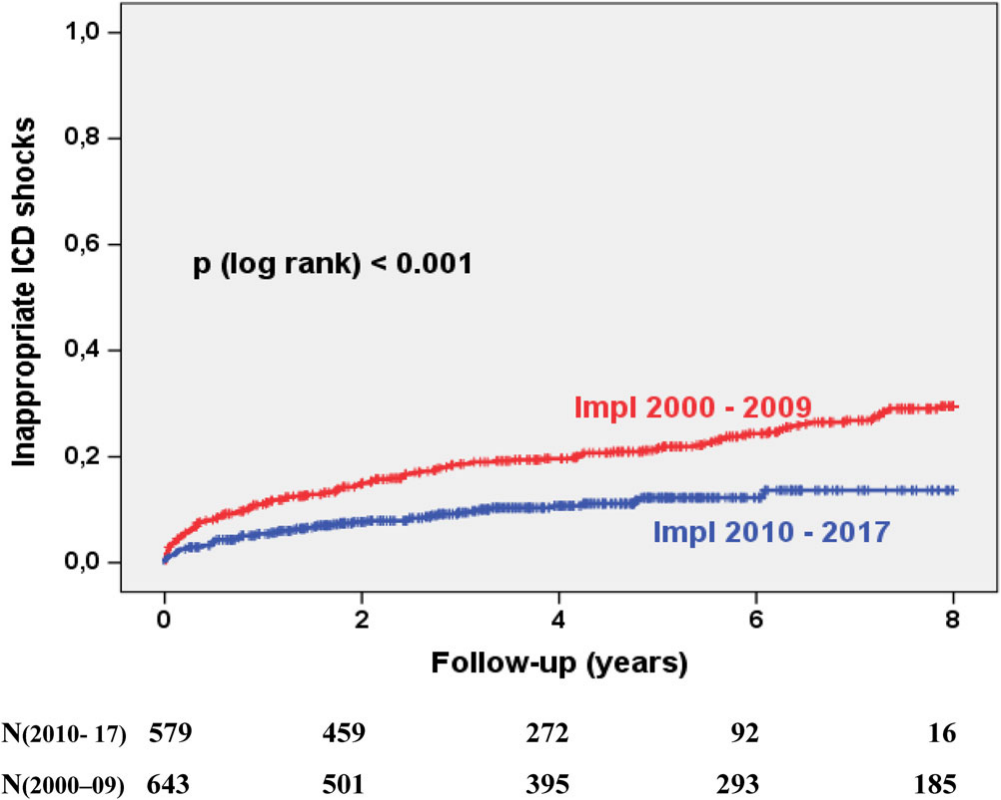
Kaplan‐Meier curves displaying the incidence of inappropriate ICD shocks. Patients were stratified according to the implantation decade. ICD, implantable cardioverter defibrillator

**Figure 4:**
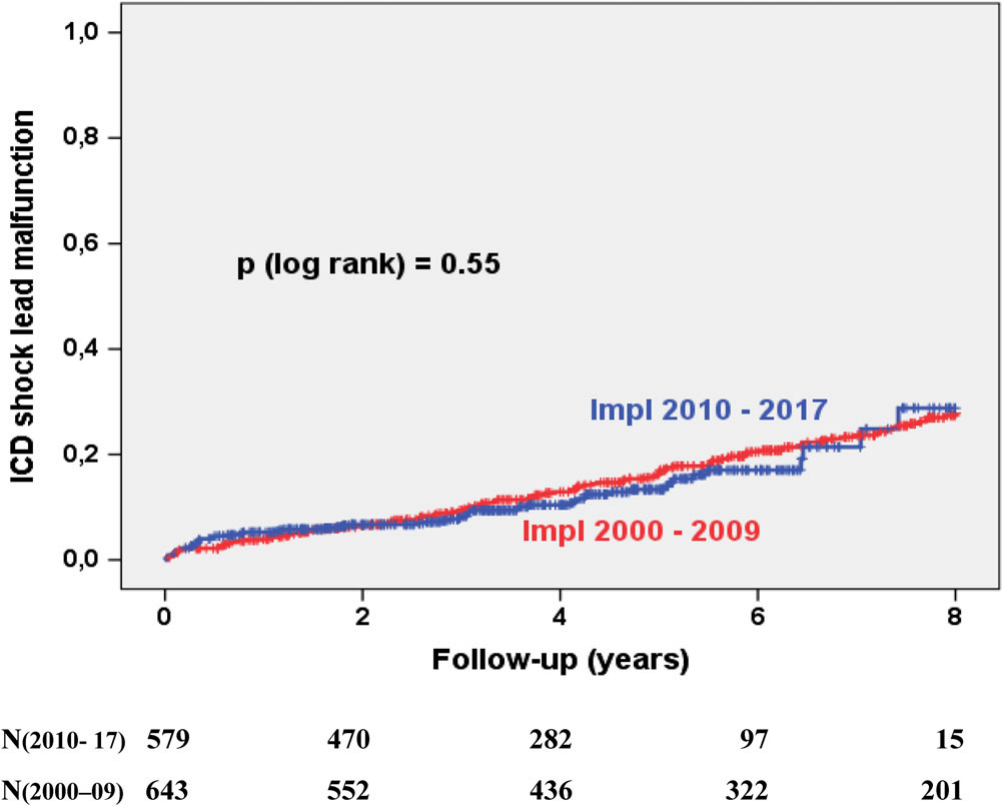
Kaplan‐Meier curves showing the incidence of ICD lead failure or malfunction. Patients were stratified according to the implantation decade. ICD, implantable cardioverter defibrillator

The overall complication rate changed slightly from 53% to 47% (*P* = .004, Figure 2). This decline was mainly driven by the reduction of inappropriate ICD shocks (30% vs 14%, *P* < .001, Figure 3). The perioperative complications of the index ICD implantation procedure remained similar with 4.8% in group 1 and 6.2% in group 2. The rate of ICD lead malfunction rate after 8 years remained high and unchanged (29% in 2010s vs 28% in 2000s, *P* = n.s., Figure 4). Causes for ICD malfunction were structural defects (isolation failure or fracture) in 75%, sensing or pacing problems in 14% and perforation/ dislocation in 11%. No change was observed with regard to the incidences of device infections after 8 years (5.2% in 2010s vs 6.7% in 2000s, *P* = n.s.), the incidence of pocket revision after 8 years (1.6% in 2010s vs 2.8% in 2000s, *P* = n.s.) or the incidence of device recalls needing revision (1% vs 0.6%, *P* = n.s.). There were 4 ICD‐related deaths in the 2000s and no ICD‐related death in the 2010s. Two patients died from device infection, one patient from superior vena cava rupture and cardiac tamponade during lead extraction due to lead failure and one patient from ventricular fibrillation induced by inappropriate shock which occurred due to artifact sensing caused by lead fracture. The induced VF could not be terminated by the ICD due to the lead fracture.

The benefit‐harm profile at the end of follow‐up was as follows: 33% of patients had benefit from ICD therapy (n = 409) and 35% ICD complications (n = 427). Twenty‐two percent (n = 264) of ICD patients had only harm from ICD without benefit. In a multivariate analysis including the factors age > 70 years, female gender, EF < 30%, nonischemic heart disease, diabetes, atrial fibrillation, CRT, Riata ICD lead and implantation decade the only independent predictor for increased incidence of ICD‐treated ventricular tachyarrhythmias was atrial fibrillation whereas implantation in 2010s was associated with a lower incidence of appropriate ICD therapy (Table 2). Independent predictors for ICD complications were atrial fibrillation and nonischemic cardiomyopathy whereas low EF < 30% and implantation in the 2010s were associated with a lower rate of ICD complications. Riata ICD lead was not associated with an increased complication rate. Nonischemic cardiomyopathy was an independent predictor for ICD complications without benefit from ICD therapy (Table 2).

**Table 2:** Cox regression analysis for predictors of ICD therapy, ICD complications, and ICD complications without benefit

	Hazard ratio	95% Confidence interval
Predictors for ICD‐therapy		
Age > 70 years	0.90	0.72‐1.13
Female	0.81	0.62‐1.07
EF < 30%	0.93	0.71‐1.22
Nonischemic CMP	0.99	0.81‐1.22
Diabetes	1.05	0.84‐1.28
Atrial fibrillation	1.39	1.14‐1.70^a^
CRT	0.92	0.75‐1.14
Riata lead	0.99	0.77‐1.24
Implantation 2010‐2017	0.63	0.50‐0.81^a^
Predictors for complications		
Age > 70 years	0.95	0.76‐1.18
Female	0.86	0.67‐1.12
EF < 30%	0.73	0.57‐0.94^a^
Nonischemic CMP	1.29	1.06‐1.57^a^
Diabetes	0.93	0.76‐1.15
Atrial fibrillation	1.32	1.08‐1.61^a^
CRT	0.95	0.78‐1.17
Riata lead	0.92	0.73‐1.17
Implantation 2010‐2017	0.74	0.59‐0.94^a^
Predictors for complications without benefit		
Age > 70 years	1.06	0.81‐1.39
Female	0.83	0.60‐1.16
EF < 30%	0.75	0.54‐1.04
Nonischemic CMP	1.37	1.07–1.77^a^
Diabetes	0.93	0.71‐1.21
Atrial fibrillation	1.03	0.79‐1.34
CRT	0.95	0.73‐1.23
Riata lead	0.98	0.71‐1.36
Implantation 2010‐2017	1.03	0.76‐1.39

CMP, cardiomyopathy; CRT, cardiac resynchronization therapy; EF, ejection fraction; ICD, implantable cardioverter defibrillator.

a *P* < .05.

## 4 DISCUSSION

### 4.1 Major findings

The ICD therapy rate for ventricular arrhythmias in patients with primary prophylactic ICD implantation is decreasing over the last two decades. The complication rate has slightly reduced due to a substantial decrease of inappropriate ICD shocks whereas ICD shock lead problems and device infection remain a major issue in ICD therapy. Nearly a quarter of primary prophylactic ICD patients has only ICD complications without receiving appropriate ICD therapy. The presence of nonischemic cardiomyopathy is an independent predictor for ICD complications without benefit of ICD therapy.

### 4.2 Benefit of ICD therapy during follow‐up

The rate of appropriate ICD therapy decreased over the last two decades. This reduction included ICD shocks, and ATP therapy as well as VT and VF episodes. One important reason might be attributed to the temporal trend of a less aggressive programming of the VT/VF zones. Several studies showed that change of ICD programming to less aggressive therapy resulted in lower mortality rates.^9^, ^10^, ^11^, ^12^ Consequently, programming of ICD was adapted during the past 15 years according to the recommendations of the randomized trials.

Another explanation for the lower rate of VT/VF episodes in the primary prophylactic ICD population might be the change of clinical characteristics towards a less severely progressed heart failure disease at ICD implantation as well as a better heart failure treatment. The 2010s primary prophylactic ICD group had less often renal failure at implantation, less often digoxin therapy and more often spironolactone compared to the 2000s group. The 8‐year mortality rate was 12% lower compared to patients implanted in the 2000s. In addition, the reduction of ICD therapy did include a reduction of VF episodes which cannot be explained only by a change of ICD programming. Our observations are supported by a previous study where Boveda et al. observed a significant change in patterns of use and outcomes in primary prevention ICD over the last decade with reductions in mortality and appropriate therapies. These reductions were counterbalanced by an increase in late ICD complications.^13^

### 4.3 Complications in ICD therapy

A substantial decrease of inappropriate ICD shocks was seen between 2000 and 2018 which can be explained partly by changes of ICD programming as mentioned above. Additionally, improved discrimination algorithm of lead noise or supraventricular tachycardias from ventricular tachyarrhythmias prevented inappropriate ICD shocks in the later implantation groups.^14^, ^15^ ICD shock lead problems remained a major problem of ICD therapy. The failure or malfunction rate was unchanged during the two decades and caused nearly half of the ICD complications. About one third of ICD leads (n = 327, 27%) were St. Jude Medical Riata™ (now Abbott) ICD leads which are known to be prone to insulation failure^16^ and had been removed from the market in 2010.^17^ Interestingly, Riata ICD leads were not associated with an increased ICD complication rate and modern ICD leads did not show a better performance than older leads. Precise analysis of lead failure mechanisms are needed to find strategies to ameliorate long‐term performance. This might include lead design as well as implantation techniques. The majority of ICD leads were implanted by subclavian puncture. Alternative accesses like cephalic or axillary vein access or implantation of subcutaneous ICDs might reduce ICD lead problems.^18^, ^19^, ^20^

The most serious ICD complication is the device infection which remained unchanged during the two decades. Standard guidelines for prevention of device infection were applied as accurate as possible.^21^ One of the strongest known predictors for device infection is a revision procedure as bacterial colonization can exacerbate during a revision procedure.^22^ Therefore revision interventions should be avoided as much as possible. Longer battery longevity, less ICD lead revisions due to lead failure and conservative management of device recalls should reduce the revision rate and consecutively the infection rate. Other forms like the use of antibacterial envelope might further reduce the infection rate.^23^

### 4.4 The benefit‐harm profile

In the present study 33% had termination of potentially malignant ventricular tachyarrhythmias at the end of follow‐up. But more important, 22% had only ICD complications including potentially life‐threatening harm without need of appropriate ICD therapy during follow‐up. Nonischemic cardiomyopathy was the only predictor for ICD complications without benefit from ICD therapy. Importantly, nonischemic cardiomyopathy was not associated with a lower benefit with regard to termination of ventricular tachyarrhythmias by the ICD. The potential benefit of ICD therapy in nonischemic cardiomyopathy is questioned at least since the publication of the DANISH trial, which showed no benefit of ICD therapy in this patient group.^5^ Other meta‐analyses emphasized the efficacy of primary prophylactic ICD therapy in nonischemic cardiomyopathy^24^, ^25^ but did not analyze complication rates. The present study shows a group of patients, which represents nearly a quarter of ICD patients among primary prophylactic ICD patients who have only harm from ICD therapy. As consequence careful selection and detailed informed consent of patients is necessary before implantation of primary prophylactic ICD implantation.

### 4.5 Study limitations

As the current study is a single‐center registry, our observations and conclusions may not be necessarily generalized. ICD related complications might dependent from local conditions like operation technique, choice of manufacturer or device programming. Especially the rate of ICD shock lead malfunctions might be influenced by certain leads having higher rates of malfunction like the Riata lead.^16^ ICDs were implanted over a long time period from 2000 to 2017, on account of that evolving and expanding guidelines for the implantation of ICDs, device programming, and pharmacological treatment of arrhythmias might have created a heterogeneous population.

## 5 CONCLUSIONS

The rate of ICD therapy for ventricular arrhythmias is decreasing while the complication rates remain high due to an unchanged elevated malfunction rate of ICD shock leads after 8 years and an unchanged device infection rate. Future efforts should focus on improving the long‐term performance of ICD shock leads and reduction of device infections. Careful selection and detailed informed consent of patients is necessary before implantation of primary prophylactic ICD implantation.

## CONFLICT OF INTEREST

The authors declare no potential conflict of interests.
